# Teachers’ Well-Being and Associated Factors during the COVID-19 Pandemic: A Cross-Sectional Study in Hong Kong, China

**DOI:** 10.3390/ijerph192214661

**Published:** 2022-11-08

**Authors:** Sam S. S. Lau, Eric N. Y. Shum, Jackie O. T. Man, Ethan T. H. Cheung, Padmore Adusei Amoah, Angela Y. M. Leung, Orkan Okan, Kevin Dadaczynski

**Affiliations:** 1 Research Centre for Environment and Human Health, School of Continuing Education, Hong Kong Baptist University, Hong Kong, China; 2 Multidisciplinary Research Centre, School of Continuing Education, Hong Kong Baptist University, Hong Kong, China; 3 College of International Education, Hong Kong Baptist University, Hong Kong, China; 4 Institute of Bioresource and Agriculture, Hong Kong Baptist University, Hong Kong, China; 5 School of Graduate Studies, Department of Applied Psychology, Institute of Policy Studies, Lingnan University, Hong Kong, China; 6 School of Nursing, Hong Kong Polytechnic University, Hong Kong, China; 7 Department of Sport and Health Sciences, Technical University Munich, 80333 Munich, Germany; 8 Public Health Centre Fulda, Fulda University of Applied Sciences, 36037 Fulda, Germany; 9 Center for Applied Health Science, Leuphana University Lueneburg, 21335 Lueneburg, Germany

**Keywords:** mental health, school teachers, workplace well-being, school closure, perceived stress, occupational health, coping, sense of coherence, Hong Kong

## Abstract

School teachers have faced many challenges due to the coronavirus disease-2019 (COVID-19) pandemic and public health-related containment measures. Recent studies have demonstrated high levels of stress and mental health issues among school teachers. To better understand teacher well-being and inform practices to support them in the face of the ongoing pandemic, we aimed to assess perceived stress, well-being and associated factors among school teachers in Hong Kong, China. For this cross-sectional study, we employed a self-reported questionnaire to assess teacher well-being as an indicator of mental health. Drawing on quantitative data obtained from 336 teachers in Hong Kong from April 2021 to February 2022, we assessed workloads, work-related sense of coherence, perceived stress, secondary burnout symptoms (i.e. intensification of work and exhaustion related to work situation), self-endangering work behaviours and satisfaction with work. Bivariate and multivariate analyses were performed to examine the associations between well-being, demographic and work characteristics. A high percentage (87.6%) of teachers had high levels of perceived stress, which was positively associated with extensification of work (*r* = 0.571, *p* < 0.01), intensification of work (*r* = 0.640, *p* < 0.01) and exhaustion related to work situation (*r* = 0.554, *p* < 0.01). A multilinear regression model adjusted for age and gender was computed to detect predictors of teachers’ well-being index values (F(12, 296) = 41.405, *p* < 0.001, R^2^ = 0.627). A higher WHO-5 score was associated with (1) higher teaching hours (B = 0.235, 95% CI = 0.093, 0.413, *p* = 0.002); (2) higher work-related sense of coherence (B = 2.490, 95% CI = 0.209, 4.770, *p* = 0.032); (3) higher work satisfaction (B = 5.410, 95% CI = 2.979, 7.841, *p* < 0.001); (4) lower level of exhaustion related to work situations (B = −9.677, 95% CI = −12.279, −7.075, *p* < 0.001); and (5) lower level of psychosomatic complaints (B = −4.167, 95% CI = −6.739, −7.075, *p* = 0.002). These findings highlight the critical need to allocate more attention and resources to improve the mental health of school teachers in Hong Kong. The findings can also inform the development of psychological and organisational interventions and support mechanisms for teachers during the prolonged COVID-19 pandemic and in preparation for future stressful scenarios. Safeguarding the well-being and mental health of teachers is important for improving the quality of teaching and learning environments and the mental health of school students.

## 1. Introduction

The coronavirus disease 2019 (COVID-19) pandemic has disrupted life and significantly negatively impacted the mental health of individuals worldwide [1]. In particular, in countries that implemented lockdowns, people have faced an increased risk of developing mental disorders, such as depression and anxiety [2,3]. School closures were also enforced by governments around the world during the first part of the pandemic [4,5], forcing millions of teachers and students to turn to remote teaching and learning. With little to no training in remote teaching, teachers were required to quickly adapt not just themselves but also their students to these new methods [6]. Video conferencing and other online resources became the new normal in the delivery of education. The technical skills of teachers in using digital tools for teaching and communication might have affected their perceptions of remote teaching, as teachers with fewer digital skills may perceive remote techniques as a burden [7,8], resulting in work-related stress [9]. Teachers must find time to attend to various online learning-related needs of students, and a key challenge faced by teachers has been to resolve the digital divide amongst students [10]. The Census and Statistics Department reported that despite the majority (94%) of households in Hong Kong having access to the Internet, only 71% of economically disadvantaged households (i.e., earning < HK$10,000 (ca. US$1282) per month) had access to the Internet [11]. Teachers also need to take care of students with special educational or mental health needs, who are less likely than student without such needs to be supported adequately when transitioning to virtual learning [12,13]. 

Before the pandemic, teaching had already been recognised as a highly stressful occupation [14,15,16]. The additional workload and expectations generated by the pandemic have heightened teachers’ stress levels and placed them at greater risk of anxiety and burnout than they were in pre-pandemic times. There is growing evidence that the forced transition to remote teaching has caused a global mental health crisis in teachers [17,18,19]. Moreover, it is clear that teachers are an occupational group severely affected by the pandemic, both professionally and personally. The sudden shift in working environment has become a new source of pressure for teachers, which has also affected their physical and mental well-being [17]. The United Nations Educational, Scientific and Cultural Organization has recognised confusion and stress as adverse effects of school closures [4], with these effects due to uncertainties, unpredictable durations of closure, a lack of clarity regarding teachers’ responsibilities, and teachers’ requirements to maintain connections with students. In addition, the use of information and communication technologies may pose challenges to the physical and psychological well-being of individuals and even to their job performance [18]. A survey of 1278 Canadian teachers revealed that the rise in expectations as they adapted to remote teaching was a major source of stress [19]. A study of 380 teachers in Germany showed that the vast majority of participants considered a lack of access to computer hardware and poor Internet connectivity as major barriers to distance teaching [20]. Teachers may also experience ‘technostress’ when they cannot adapt to or cope with digital communication and information technologies in a healthy manner. Technostress has been associated with psychological and behavioural disorders and could reduce job satisfaction, work commitment and job performance while intensifying negative feelings such as anxiety, worry and negative self-view [21]. Technostress is also a psychological reaction that negatively affects university teachers’ work and significantly impacts more on older teachers [22,23,24]. In addition, a longitudinal study of in England showed that the prevalence of anxiety in teachers peaked before school closures and re-openings [25]. During such challenging periods, symptoms of stress, anxiety and depression were reported by many Portuguese teachers [26]. Teacher burnout was found to be linked to both pandemic anxiety and a lack of administrative assistance [27]. Characterised by the three dimensions of exhaustion, cynicism and inefficacy, burnout is a sustained reaction to persistent interpersonal and emotional pressures at work [28] and can be assessed using the dimensions of exhaustion as well as psychosomatic complaints [29]. Although many countries are concerned about their teachers’ mental health and well-being, little is known about factors associated with teachers’ mental health and well-being during the pandemic in Hong Kong, especially with respect to the implementation of stringent health preventive measures in the context of a dynamic ‘zero-COVID’ policy [30]. Thus, it is important to assess the level of stress and burnout experienced by teachers in Hong Kong during the pandemic period. 

Work-related sense of coherence (work-SOC) refers to ‘the perceived comprehensibility, manageability and meaningfulness of an individual’s current work situation’ and consists of three dimensions: comprehensibility, manageability and meaningfulness [31,32]. Comprehensibility describes the degree to which people perceive their workplaces as organised, consistent and clear [33]. Manageability is an instrumental concept and describes the extent to which an individual believes that there are sufficient resources available to meet the demands made by the workplace [34]. Meaningfulness is a motivational component indicating whether the work environment is perceived as deserving of dedication and engagement [34]. In the context of COVID-19, a recent study of healthcare professionals in Switzerland revealed that professionals with higher levels of work-SOC reported lower levels of COVID-19-related anxiety, psychological trauma, depression and perceived vulnerability [35]. Although work-SOC has been suggested to protect healthcare professionals from developing symptoms of psychological illness, the pandemic appears to be eroding this defence [35]. Similarly, a study of non-health workers in Spain revealed that participants with the lowest levels of SOC had the highest percentages of distress [36]. Thus, the level of work-SOC could be a critical determinant of the mental health of an individual. However, studies of work-SOC among educators have been limited, especially in Asian countries. Therefore, it is important to examine the association between the level of work-SOC and the well-being of teachers in Hong Kong during the pandemic. 

When workers are confronted with large workloads and strong demands for self-organisation, they may resort to self-endangering work behaviours, i.e., ‘behaviours that may be functional with regard to attaining work goals but dysfunctional with regard to health and long-term ability to work’ [37]. This was originally proposed by Dettmers et al. in the context of the stress transformational mode [37] as a coping mechanism to treat diminished well-being brought on by heavy workloads and demanding expectations of self-management. A recent study of the Japanese general population revealed that self-endangering work behaviours were more prevalent in cases of flexible work schedules and long working hours than for rigid schedules and short working hours [38]. However, noting the forced transition to remote teaching made teachers’ work schedules more flexible and increased working hours, it is important to explore the prevalence of self-endangering work behaviours among teachers in Hong Kong during the pandemic.

### 1.1. Teachers in Hong Kong

Teachers in Hong Kong experienced several frequent changes to their work environments during the pandemic. School lockdowns were first implemented in January 2020 for approximately 4 months. During the third and fourth waves of the pandemic, a hybrid mode of teaching was followed, with schools offering half-day learning at school sites and additional online classes at home. Schools were fully closed again during the fifth wave. Clearly, these frequent changes in response to the perceived severity of the pandemic caused confusion and considerable uncertainty among teachers and school staff. Uncertainties can cause stress that can in turn affect the well-being of individuals by challenging their capacity to predict, plan and act efficaciously [39,40]. Teacher well-being is a critical factor in determining teaching effectiveness and students’ academic performance and well-being [41,42,43,44]. Well-being refers to healthy and successful functioning of teachers at work [45]. Regarding the well-being and mental health status of teachers in Hong Kong, a survey in November 2020 revealed that more than 80% of teachers had had their physical and psychological health negatively impacted by the pandemic or the government’s health preventive responses to the pandemic and 85% of them considered their work pressure to be high [46,47]. The perceived stress was significantly and positively correlated with their working hours. The majority of teachers reported their major work stressors to be online teaching (78%), recovery of teaching progress (72%) and the widening learning gaps in students (66%). Nearly half of the teachers reported feeling anxious (48%), and the majority reported feeling exhausted (85%) and disappointed (59%). 

### 1.2. Aims and Objectives

A large proportion of pandemic research on mental health and well-being in Hong Kong has been conducted mainly in healthcare workers [48,49,50,51] with limited studies in students [52,53]. To the best of our knowledge, this is the first community-wide study to examine teachers’ well-being and stress during the COVID-19 pandemic following multiple school closures in Hong Kong. We aimed to explore the association of the pandemic and the government’s health preventive responses to it with the working lives, mental health and well-being of Hong Kong school teachers using quantitative survey methods. Thus, we examined self-endangering work behaviours, perceived stress, and work-SOC and their associations with teachers’ well-being. Our findings increase understanding of the stress level and mental health status of teachers and could inform the development of individual and organisational support mechanisms for teachers. 

## 2. Materials and Methods

### 2.1. Study Design, Participants and Procedure

We adopted a descriptive cross-sectional design. This study was conducted as part of an international study of School Health Literacy based on the COVID Health Literacy Network, a global research network that comprises more than 150 researchers from 70 countries (https://covid-hl.eu) (accessed on 2 October 2022). We adapted a questionnaire from Dadaczynski, Okan and Messer [54] by translating it from English to traditional Chinese and modifying the translated version to suit the conceptual, cultural and linguistic settings in Hong Kong. The pre-final version in Chinese was then reviewed by the first, third, fourth and sixth authors and rephrased based on feedback collected from a pilot study with eight participants. 

The study sample consisted of teachers working in primary, secondary and special schools in Hong Kong. Data were collected from April 2021 to February 2022 using purposive convenience sampling, Invitations were sent to the principals of 1,130 schools (561 primary schools, 477 secondary schools and 36 special schools) registered with the Education Bureau in Hong Kong. An online self-report questionnaire was launched on the Qualtrics platform, in both English and Chinese language versions, and the link was distributed via email. Participants were also recruited through authors’ personal networks via social media platforms (e.g., WhatsApp). In addition, hardcopy questionnaires were distributed to 243 school principals in our established school networks through postal mail with a priori verbal agreement obtained from the schools by telephone. Participants were requested to invite other eligible participants to take part in the study while maintaining anonymity. Brief information on the purpose, benefits, risks and confidentiality of the study was given on the first page of the questionnaire and informed consent was obtained from the participants. Participation was anonymised to avoid potential bias. No incentives were provided to the participants for completing the questionnaire. The online questionnaire was set such that participants could withdraw at any time if they wished to. The eligible sample for analysis comprised 366 teachers (53.6% women and 46.4% men). Their ages ranged from 20 to 65 years (mean [M] = 38.3 years, standard deviation [SD] = 9.72). 

### 2.2. Measures

A self-administered questionnaire was used to gather information on demographics, work-related factors (e.g., working hours, satisfaction), work-SOC, perceived stress, self-endangering work behaviours and secondary burnout symptoms.

### 2.3. Demographic Profile

The demographic details collected were age (in years), gender (male vs. female) and school type (primary, secondary or special).

### 2.4. Work-Related Factors

Participants were questioned regarding their weekly teaching hours, weekly working hours, adjustments to weekly working hours due to the COVID-19 pandemic and the number of students enrolled in their respective schools. Work satisfaction was assessed by asking respondents to rate their overall satisfaction with their jobs on a 5-point scale, ranging from 1 for very dissatisfied to 5 for very satisfied.

### 2.5. Health-Related Factors

#### 2.5.1. Well-Being

According to World Health Organization, mental health refers to a “state of well-being in which the individual realizes his or her own abilities, can cope with the normal stresses of life, can work productively and fruitfully, and is able to make a contribution to his or her community” [55]. Wellbeing comprises “an individual’s experience of their life as well as a comparison of life circumstances with social norms and values” [56]. The well-being of teachers in this study was measured using the World Health Organization Well-being Index [57], which is among the most widely used questionnaires assessing subjective psychological well-being [58]. The scale consists of five Likert-type statements requiring responses on a 6-point scale, from ‘never’ to ‘always’. Participants were asked to rate the frequency at which they experienced the feeling described in the statements during the 2 weeks preceding the survey. The well-being score was calculated by adding the scores for the five items and multiplying the answer by 4; higher scores signified a higher level of well-being. A score of ≤50 indicates poor wellbeing and a score of ≤28 is indicative of depression [58]. 

#### 2.5.2. Stress

Perceived stress at work was measured using the 10-item Perceived Stress Scale (PSS-10 [59]). This scale consists of 10 Likert-type questions requiring responses on a 5-point scale, from ‘never’ to ‘very often’. The scale was adapted to the COVID-19 context and its wording was modified for a school context. The total score was calculated by reversing the scoring of items 4, 5, 7 and 8 and adding the sum of all 10 items. A higher score indicates a higher perception of stress. The PSS-10 has been shown to have high content and construct validity [60,61,62].

#### 2.5.3. Work-SOC

Work-SOC was assessed using a 9-item scale [63], consisting of three subscales: comprehensibility (four items), manageability (two items) and meaningfulness (three items). Previous research has shown that the overall scale has very good reliability (Cronbach α = 0.83) and that the subscales have acceptable to good reliability (Cronbach α = 0.72 to 0.84) [33]. Participants gave ratings on 7-point semantic differentials, each with bipolar adjective pairs (e.g., ‘manageable’ vs. ‘not manageable’). Mean scores of the items were calculated as total scores, with higher scores indicating a stronger work-SOC. The Cronbach α of the overall scale in the present study was 0.764. 

#### 2.5.4. Stress-Related Self-Endangering Work Behaviours

Stress-related self-endangering work behaviours were measured using three subscales of the self-endangering work behaviour scale [37]: extensification of work, intensification of work and quality reduction. The latter two subscales each have three items, whereas the first subscale has six items, all of which are Likert-type questions requiring responses on a 7-point scale from ‘never/very rarely’ to ‘very often’. The total score for each subscale was calculated by averaging the item scores in each subcategory and dividing the result by the total number of items. A greater score indicates a higher level of work extensification, intensification or quality decrease. Previous research revealed that the extensification of work subscale has very good reliability (Cronbach α = 0.81) and that the intensification of work subscale has excellent reliability [37].

#### 2.5.5. Burnout Symptoms

Exhaustion related to work situation was measured by the ‘exhaustion’ subscale of the Burnout Assessment Tool (BAT) [29]. The subscale consists of three Likert-type questions requiring responses on a 5-point scale, from ‘never’ to ‘always’. The total exhaustion score was calculated by dividing the sum of item scores by the total number of items. Higher scores indicate greater exhaustion. Previous research found that the subscale has satisfactory reliability (Cronbach α = 0.85–0.87) [29]. In addition, psychological discomfort was measured by the ‘psychosomatic complaints’ subscale of the BAT [29]. The subscale consists of five Likert-type questions requiring responses on a 5-point scale, from ‘never’ to ‘always’. The total score was calculated by adding and dividing the item scores by the number of items and ranged from 1 to 5. Higher scores indicate more psychosomatic complaints. According to the statistical norm reported by Schauefeli et al. [29], the total score for exhaustion and psychosomatic complaints were divided into four levels: low, average, high and very high. 

### 2.6. Ethics Statement

This study was approved by the Hong Kong Baptist University’s Research Ethics Committee (REC/20-21/0465). All of the participants were informed of the study objectives, procedures, data collection, anonymisation and confidentiality of all personal data. All of the participants provided informed consent and were informed that they could withdraw at any time from the study. 

### 2.7. Statistical Methods

We performed calculations and analysis of the survey data using SPSS 27.0 (IBM Corp. Released 2020; IBM SPSS Statistics for Windows, Version 27.0; IBM Corp., Armonk, NY, USA). The data were anonymised and checked by the research team. Descriptive analyses were performed to determine the demographic and work-related characteristics of the sample. Stress, stress-related behaviours and well-being were also recorded. Descriptive statistics are reported as means (M), standard deviations (SD), and percentages (%). Bivariate Pearson correlation analyses were conducted to examine interrelations between key variables. The results are displayed as Pearson correlation coefficients (*r*). Independent sample *t*-tests and analyses of variance (ANOVAs) were conducted to determine differences between the level of perceived stress and well-being of each demographic group. The level of statistical significance was set as a two-sided *p* < 0.05.

## 3. Results

The questionnaire was completed by 366 teachers with a mean age of 38.3 years (SD 9.72), an age range of 20 to 65 years, and 53.6% female. There was no significant difference in age between the male (M = 38.7) and female (M = 38.0) participants (*p* = 0.845). Table 1 reveals the respondents’ demographic profile, workload and work-related characteristics. Nearly half (45.1%) of the participants worked at secondary schools, a third (33.8%) in primary schools and approximately one-fifth (21.1%) in special schools. The average weekly working hours and teaching hours were 45.4 (SD = 16.18) and 21.9 (SD = 10.15) h, respectively. Slightly more than half (51.2%) of the participants worked longer weekly hours during the COVID-19 pandemic than before the pandemic. 

Table 2 illustrates descriptive statistics of participants’ well-being, stress and related behaviours, work-SOC and physical health. The total mean scores of the WHO-5, perceived stress and work-SOC were 50.3 (SD = 21.3), 31.1 (SD = 4.22), and 4.71 (SD = 0.85), respectively. With regard to self-endangering work behaviours, the total mean score of extensification of work was 3.36 (SD = 0.77), whereas those of intensification of work and quality reduction were 3.09 (0.88) and 2.40 (0.94), respectively. In terms of work satisfaction, over half (51.5%) of the participants reported being ‘neither satisfied nor dissatisfied’ with their jobs. 

Table 3 shows the correlations between the key variables. Perceived stress was strongly and positively correlated with extensification of work (*r* = 0.571, *p* < 0.01), intensification of work (*r* = 0.640, *p* < 0.01), and exhaustion related to work situation (*r* = 0.554, *p* ≤ 0.01). In addition, perceived stress was positively correlated with working hours, perceived general health, quality reduction and psychological complaints (*r* = 0.210–0.350, *p* < 0.01). In contrast, perceived stress was negatively correlated with WHO-5 score (*r* = −0.465, *p* < 0.01), work satisfaction (*r* = −0.406, *p* < 0.01) and work-SOC (*r* = −0.271, *p* < 0.01).

WHO-5 score was positively correlated with work-SOC (*r* = 0.449, *p* < 0.01) and strongly negatively correlated with extensification of work (*r* = −0.524, *p* < 0.01), intensification of work (*r* = −0.519, *p* < 0.01), exhaustion related to work situation (*r* = −0.522, *p* < 0.01) and psychosomatic complaints (*r* = −0.522, *p* < 0.01). WHO-5 score was also negatively related to work satisfaction (*r* = −0.482, *p* < 0.01), working hours (*r* = −0.243, *p* < 0.01), and quality reduction (*r* = −0.264, *p* < 0.01). 

The participants were further divided into groups based on their age, gender, type of school, level of exhaustion related to work and psychosomatic complaints. *T*-tests and ANOVAs were performed to identify the mean between-group differences in the total mean scores on the WHO-5 and perceived stress (Table 4). No significant differences in WHO-5 score (*p* = 0.065) and perceived stress (*p* = 0.089) were detected between males and females. Moreover, no significant differences in WHO-5 score (*p* = 0.086) and perceived stress were detected between different age groups (*p* = 0.816). Regarding the type of school, a significant difference was detected in the level of perceived stress (*p* < 0.001), but not in WHO-5 score (*p* = 0.646). Participants who worked at special schools had a significantly higher level of well-being than those who worked at primary and secondary schools. Participants with high or very high levels of exhaustion related to work scored significantly lower on the WHO-5 (*p* < 0.001) and higher in perceived stress (*p* < 0.001) than those with average to low levels of exhaustion related to work. Regarding psychosomatic complaints, teachers with high or very high levels of psychosomatic complaints scored significantly lower on the WHO-5 (*p* < 0.001) and higher in perceived stress (*p* < 0.001) than those with low levels of psychosomatic complaints. 

Table 5 presents the regression model for predicting the teachers’ WHO-5 score. A multilinear regression model adjusted for age and gender was computed for detecting these predictors (*F*(12, 296) = 41.405, *p* < 0.001, *R*^2^ = 0.627). A higher WHO-5 score was associated with (1) higher teaching hours (*B* = 0.235, 95% CI = 0.093, 0.413, *p* = 0.002); (2) a higher work-SOC (*B* = 2.490, 95% CI = 0.209, 4.770, *p* = 0.032); (3) higher work satisfaction (*B* = 5.410, 95% CI = 2.979, 7.841, *p* < 0.001); (4) a lower level of exhaustion related to work situations (*B* = −9.677, 95% CI = −12.279, −7.075, *p* < 0.001); and (5) a lower level of psychosomatic complaints (*B* = −4.167, 95% CI = −6.739, −7.075, *p* = 0.002).

Table 6 illustrates the regression model use for predicting teachers’ perceived level of stress. The multilinear regression model was adjusted for age and gender (*F*(12, 296) = 31.165, *p* < 0.001, *R*^2^ = 0.558). The model revealed that teachers’ level of perceived stress was positively associated with (1) lower age (*B* = −0.018, 95% CI = −0.026, −0.010, *p* < 0.001); (2) higher scores for work intensification (*B* = 1.733, 95% CI = 1.158, 2.307, *p* < 0.001); (3) work extensification (*B* = 0.897, 95% CI = 0.260, 1.499, *p* = 0.006); and (4) exhaustion related to work situation (*B* = 0.961, 95% CI = 0.353, 1.570, *p* = 0.002). 

## 4. Discussion

### 4.1. Teachers’ Stress

This study aimed to understand the mental health and well-being of school teachers in Hong Kong during the COVID-19 pandemic and the impacts of these factors on schooling. We found that teachers in Hong Kong have a high level of perceived stress, with the majority of participants (87.6%) perceiving that they were under a high level of stress. This is similar to the results of a survey conducted by the Hong Kong Federation of Education Workers (2021), wherein 85% of teachers felt that their work stress was too high. We found that the total mean score for perceived stress was 31.1 (SD = 4.22), which was much higher than that reported in a recent study of Taiwanese school principals’ well-being that adopted the same scale (12.7 ± 4.5) [64]. Compared with a study of teachers’ stress during the Severe Acute Respiratory Syndrome (SARS) epidemic in Hong Kong in 2003 [65], the mean of measure stress was 34.83 (out of 48), recorded using the 20-item Teacher Stressor Scale. Thus, the teachers’ responses to our questionnaire indicated that their perceived stress was proportionally higher than that recorded in teachers during the SARS epidemic in 2003. A similar study on teachers’ stress in Romania during the COVID-19 pandemic reported a mean perceived stress of 2.35 (out of 5) using the 14-item Perceived Stress Scale [66], which was lower than that measured in our study. Our findings are comparable with the key results presented in a recent systematic review [67], in which the rate of reported stress symptoms ranged from 8.1% to 81.9% in studies of the general population in China, Spain, Italy, Iran, the United States, Turkey, Nepal, and Denmark during the first 6 months of the COVID-19 pandemic. 

Our findings suggest that teachers’ perceived stress does not vary based on demographic factors such as age and gender. However, studies have demonstrated the role of gender in the extent of work stress. Klassen and Chiu [68] studied 1,430 in-service teachers in western Canada and revealed that female teachers reported more workload- and classroom-related stress than male teachers. Similarly, other studies have suggested that females perceive slightly higher levels of stress than males in school settings [69,70]. Moreover, at the general population level, females were more often associated with higher stress levels than males during the first period of the COVID-19 pandemic [71]. Our finding that there was no gender effect on teachers’ stress during the study period indicates that the working conditions during this part of the pandemic were stressful for educators regardless of gender. In terms of age, our study finding is at odds with a recent systematic review [67], which suggested that younger age groups were more often associated with higher stress levels than the elderly at the general population level during the first 6 months of the pandemic. In the context of teachers and schooling communities, previous findings on the effect of age on teacher stress level have been mixed. A study of school teachers in Greece [72] revealed that older teachers experienced higher levels of stress due to a lack of support received from the government. Thus, more studies are needed to elucidate the gender and/or age effect on teacher stress, as these effects may be compounded by cultural and contextual factors [73,74], the severity of the pandemic and associated measures across different countries. 

Our study showed that participants’ perceptions of stress differed depending on the type of schools in which they work (*p* < 0.001). Compared with participants who worked at primary and secondary schools, participants who worked at special schools reported significantly higher levels of stress. Few studies of Hong Kong populations have examined differences in teacher stress levels across mainstream and special schools. However, studies in other countries have demonstrated contradictory results of mainstream primary school teachers reporting higher levels of stress than special school teachers [75,76]. When we compared only primary and secondary schools, we found no significant differences between the participants’ perceived levels of stress. We thus corroborated previous findings (e.g., [77]), which stated that the occupational stress experienced by primary and secondary school teachers did not significantly differ with the type of school (public and private schools). However, contrasting results have been reported by other studies conducted in Malaysia and Thailand, wherein secondary school teachers perceived more overall stress than their primary school counterparts [78,79]. In terms of well-being, an empirical study in China showed results contradictory to those of our current study. This previous study found that the professional well-being of Chinese special education teachers was lower than that of regular school teachers [80]. Another study showed that psychological problems amongst special education teachers resulted in higher levels of burnout and lower levels of well-being compared to these levels in other teachers [81]. These findings suggest that the stress levels experienced by regular and special school teachers differ across countries and time. Therefore, it is necessary to examine the well-being and stress levels of teachers in Hong Kong over time to determine how to improve their experience at work and maintain the quality of our education system. 

### 4.2. Teachers’ Stress and Associated Factors

Our findings revealed that work intensification (*p* = 0.006), work extensification (*p* < 0.001) and exhaustion related to work situation (*p* = 0.002) are predictors of teachers’ perceived stress. In terms of self-endangering work behaviours (i.e. work intensification and extensification), our findings are in line with a study of Japanese employees, which suggested that burnout was caused directly by workaholism or through self-endangering work behaviours [38]. Self-endangering work behaviours can be viewed as a coping mechanism when workers are challenged with large workloads and significant needs for self-organisation [37]. During the pandemic, increased workloads, work demands and working hours have been reported by teachers and educators around the world [19,66]. Teachers have been required to immediately transition to remote teaching, which became a major stressor in their work [19,82]. Teachers also experienced changes to their working environments; corresponding research revealed that flexible work schedules are associated with self-endangering work behaviours and burnout [38]. Therefore, we posit that self-endangering work behaviours, comprising work intensification and extensification, are two predictors of teachers’ perceived stress during the pandemic in Hong Kong.

With regard to exhaustion related to work situation, emotional exhaustion is a major dimension of burnout [83,84]. Burnout itself has been considered a consequence of long-term occupational stress [85]. The indivisible relationship between exhaustion and work stress has been reported by many studies (e.g., [86]). Thus, it has been suggested that exhaustion related to work situation could be a predictor of teachers’ perceived stress. 

Surprisingly, our sample did not show significant correlations between general perceived health and perceived stress. A recent study by Ervasti et al. [87] showed a decrease in self-rated health and an increase in job strain among teachers working from home during the pandemic in Finland. This could be explained by the skewness of our sample in the perceived general health score (M = 2.65).

### 4.3. Teachers’ Well-Being and Associated Factors

Our findings demonstrated that teaching hours, work-SOC and work satisfaction are positive predictors of teachers’ well-being, whereas exhaustion related to work situation is a negative predictor of their well-being. In terms of working hours, our findings are consistent with a recent study conducted in Japan, which reported an association between longer working hours and the psychological stress responses of the teachers [88]. Despite the growing literature on the relationship between workload and teachers’ well-being [89,90], the relative importance of workload, including working and teaching hours, work position and school settings, has rarely been emphasised. A previous meta-analysis highlighted the limited number of longitudinal studies examining the consistency of working long hours over time and its relationship with well-being [91]. The same study showed that the effects of working long hours are complex as they may vary significantly across working populations and depend on gender, age, working circumstances and other factors [91]. In the present study, teaching hours were weakly but positively related to well-being. Studies have suggested that certain kinds of teaching workload, such as marking and data entry, may be perceived more negatively than others [90,92]. In the context of COVID-19, many studies have revealed online teaching to be a prominent stressor for teachers. Surprisingly, though, we found that teaching hours were positively correlated with WHO-5 score, which warrants further investigation.

Our findings show that work satisfaction is a positive predictor of teachers’ well-being. This is in agreement with a previous study of the general population, which indicated that work satisfaction is a factor that influences psychological well-being [93]. However, teachers’ subjective well-being is now considered a dynamic concept that includes self-efficacy (psychological functioning), work satisfaction and income satisfaction (cognitive dimension) [94]. Thus, the present study validated that work satisfaction is a significant predictor of teachers’ well-being. However, a more comprehensive picture can be captured when other aspects are included in the assessment.

### 4.4. Limitations and Future Studies 

The findings from this study must be placed in the context of its limitations. First, the cross-sectional design allowed us to infer associations between the variables under investigation but not the directions of the associations or cause-and-effect correlations. Thus, future studies should be longitudinal in nature to determine the direction of the associations. Future studies could also use qualitative methodologies to explore the reasons for effect of the variables examined in this study on well-being and stress in school teachers. Second, we used a convenience sample that prevents our findings being generalisable. Third, the study sample was mostly participants (>95%) who completed the questionnaire in Chinese, which may limit the applicability of our results to English-speaking teachers in Hong Kong. Finally, our use of a self-report questionnaires was based on the assumption that all participants gave honest responses. Thus, the data may have suffered from response biases, such as social desirability. The study may also be limited by a sample biased towards participants who were motivated or willing to participate. Future work should reduce such biases by using multiple quantitative and qualitative methods for data collection.

## 5. Conclusions

Our study revealed that a vast majority of school teachers in Hong Kong experienced a high level of perceived stress during the pandemic. This constitutes timely evidence on the impacts of the pandemic and the government’s health preventive responses to it on school teachers and is a significant contribution to the literature on teacher well-being and stress. In particular, our study contributes to the limited literature on school teachers’ well-being and stress in Hong Kong, providing essential baseline information that can inform policies and school administrative practices aimed at reducing teachers’ levels of stress. In addition, the findings can inform the development of psychological (e.g. developing stress management programmes, like mindfulness-based interventions) and organisational interventions (e.g. redesigning work, establishing flexible work schedules and redesigning the work environment) for promoting teachers’ health and work performance [95,96,97]. Safeguarding the well-being and mental health of teachers is important for improving the quality of teaching and learning environments and the mental health of school students (e.g., [98]). Harding et al. revealed that better teacher wellbeing is associated with better student wellbeing and with lower student psychological difficulties [98]. As the COVID-19 pandemic continues to unfold in Hong Kong and across the world, the mental health and well-being of school teachers remain a public health concern. Therefore, further research is warranted on the effects of the pandemic and governments’ pandemic response on teachers’ well-being. 

## Figures and Tables

**Table 1:** Respondents’ demographic profiles, workload and work-related characteristics (*N* = 366).

Variable		*N* (%)
Gender	Male	169 (46.4)
	Female	195 (53.6)
School Types	Primary School	123 (33.8)
	Secondary School	164 (45.1)
	Special School	77 (21.2)
Number of students at school	≤500	174 (47.5)
	501–700	71 (19.4)
	701 or above	121 (33.1)
Weekly working hours	Lower than before the COVID-19 pandemic	46 (12.7)
	Approximately unchanged	130 (36.0)
	Higher than before the COVID-19 pandemic	185 (51.2)
	**Number (*N*)**	**Mean (SD)**
Age	335	38.3 (9.72)
Number of students at school	349	516.2 (315.9)
Weekly working hours	358	45.4 (16.18)
Weekly teaching hours	356	21.9 (10.15)

**Table 2:** Descriptive statistics of participants’ well-being, stress and stress-related behaviours (*N* = 366).

Variable		Number (*N*)	Mean (SD)
Well-being score		360	50.3 (21.3)
Perceived stress		363	31.1 (4.22)
Work-related sense of coherence		359	4.71 (0.85)
Self-endangering work behaviours	Extensification of work	356	3.36 (0.77)
	Intensification of work	362	3.09 (0.88)
	Quality reduction	360	2.40 (0.94)
Burnout Assessment Tool	Exhaustion related to work situation	363	3.35 (0.91)
	Psychosomatic complaints	362	2.55 (0.72)
			**%**
Work satisfaction	Very dissatisfied/quite dissatisfied	92	22.5
	Neither satisfied nor dissatisfied	188	51.5
	Very satisfied/quite satisfied	95	26

**Table 3:** Intercorrelations between key variables.

Variable	2	3	4	5	6	7	8	9	10	11	12
1. Age	−0.097	−0.109 *	−0.287 **	−0.039	0.076	0.08	−0.187 **	−0.137 *	−0.022	−0.118 *	−0.030
2. Working hours	-	−0.030	0.210 **	−0.088	−0.243 **	−0.077	0.331 **	0.187 **	−0.088	0.235 **	0.192 **
3. Teaching hours		-	0.087	0.177 **	0.154 **	0	−0.072	0.088	−0.308 **	−0.050	0.024
4. Perceived stress			-	−0.271 **	−0.465 **	−0.406 **	0.571 **	0.640 **	0.189 **	0.554 **	0.350 **
5. Work-related sense of coherence				-	0.449 **	0.467 **	−0.267 **	−0.340 **	−0.479 **	−0.341 **	−0.282 **
6. Well-being score					-	0.562 **	−0.524 **	−0.519 **	−0.264 **	−0.710 **	−0.522 **
7. Work satisfaction						-	0.359 **	0.268 **	0.329 **	0.429 **	0.411 **
8. Extensification of work							-	0.663 **	0.219 **	0.588 **	0.360 **
9. Intensification of work								-	0.254 **	0.624 **	0.474 **
10. Quality reduction									-	0.173 **	0.126 *
11. Exhaustion related to work situation										-	0.557
12. Psychosomatic complaints											-

* *p* < 0.05; ** *p* < 0.01.

**Table 4:** Well-being score and perceived stress.

Variable		Well-Being Score		Perceived Stress	
		Mean (SD)	*p*-Value	Mean (SD)	*p*-Value
Gender	Male (*N* = 167)	52.6 (22.1)	0.065	30.7 (4.13)	0.089
	Female (*N* = 192)	48.5 (20.4)		31.5 (4.29)	
Age	35 or below (*N* = 148)		0.086	20.8 (1.17)	0.816
	36 to 45 (*N* = 104)			22.5 (2.23)	
	46 or above (*N* = 105)			20.7 (2.05)	
Type of schools	Primary school (*N* = 121)	31.4 (3.72)	0.646	46.1311	< 0.001 **
	Secondary school (*N* = 163)	31.2 (4.19)		47.9500	
	Special school (*N* = 77)	30.9 (4.38)		61.3158	
Exhaustion related to work	Very high/high (*N* = 254)	42.8 (19.1)	< 0.001 **	32.2 (3.92)	< 0.001 **
	Average/low (*N* = 105)	68.4 (14.2)		28.5 (3.72)	
Psychosomatic complaints	Very high/high	37.5 (15.8)	< 0.001 **	32.7 (3.97)	< 0.001 **
	Average/low	58.3 (20.4)		30.1 (4.07)	

** *p* < 0.01.

**Table 5:** Regression model for predicting teachers’ well-being score.

Variable	*B* (95% CI)	Standardised Coefficients (β)	*p*-Value
Gender	−0.101 (−3.139, 2.937)	−0.002	0.948
Age	0.000 (−0.038, 0.038)	0.000	0.996
Teaching hours	0.253 (0.093, 0.413)	0.124	0.002 *
Working hours	−0.084 (−0.185, 0.017)	−0.063	0.105
Perceived stress	−0.039 (−0.562, 0.484)	−0.008	0.883
Work-related sense of coherence	2.490 (0.209, 4.770)	0.099	0.032*
Work intensification	−0.949 (−3.725, 1.826)	−0.040	0.501
Work extensification	−1.237 (−4.103, 1.630)	−0.046	0.397
Quality reduction	−0.678 (−2.626, 1.270)	−0.031	0.494
Exhaustion related to work situations	−9.677 (−12.279, −7.075)	−0.411	<0.001 **
Psychosomatic complaints	−4.167 (−6.739, −1.594)	−0.141	0.002 *
Work satisfaction	5.410 (2.979, 7.841)	0.204	<0.001 **

* *p* < 0.05; ** *p* < 0.01.

**Table 6:** Regression model for predicting teachers’ level of perceived stress.

Variable	*B* (95%CI)	Standardised Coefficients (β)	*p*-Value
Gender	0.236 (−0.428, 0.900)	0.028	0.485
Age	−0.018 (−0.026, −0.010)	−0.178	<0.001 **
Teaching hours	0.032 (−0.003, 0.067)	0.078	0.077
Working hours	0.001 (−0.021, 0.023)	0.004	0.931
Well-being index (WHO-5)	−0.002 (−0.027, 0.023)	−0.009	0.883
Work-related sense of coherence	−0.084 (−0.587, 0.418)	−0.017	0.742
Work intensification	1.733 (1.158, 2.307)	0.362	<0.001 **
Work extensification	0.897 (0.260, 1.499)	0.162	0.006 *
Quality reduction	0.140 (−0.286, 0.566)	0.031	0.519
Exhaustion related to work situations	0.961 (0.353, 1.570)	0.203	0.002 *
Psychosomatic complaints	−0.199 (−0.771, 0.373)	−0.033	0.493
Work satisfaction	−0.383 (−0.930, 0.164)	−0.072	0.170

* *p* < 0.05; ** *p* < 0.01.

## Data Availability

The data of the present study are available from the corresponding author upon reasonable request.
